# Supplementary material for: Physical activity, fear avoidance beliefs and level of disability in a multi-ethnic female population with chronic low back pain in Suriname: A population-based study
Source: PLoS One. 2022 Oct 31;17(10):e0276974. doi: 10.1371/journal.pone.0276974 (PMC9621425; doi:10.1371/journal.pone.0276974)
Supplement: Attachment — Submitted filename: Response to reviewers.docx [file pone.0276974.s001.docx]

**Manuscript ID:** PONE-D-21-38825

**Physical activity, fear avoidance beliefs and level of disability in a multi-ethnic female population with chronic low back pain in Suriname: a population-based study**

**Comments Academic Editor, PLOS ONE**

Dear Dr. Ho-A-Tham,

Thank you for submitting your manuscript to PLOS ONE. After careful consideration, we feel that it has merit but does not fully meet PLOS ONE’s publication criteria as it currently stands. Therefore, we invite you to submit a revised version of the manuscript that addresses the points raised during the review process.

Please submit your revised manuscript by Jul 16 2022 11:59PM. If you will need more time than this to complete your revisions, please reply to this message or contact the journal office at [plosone@plos.org](mailto:plosone@plos.org). When you're ready to submit your revision, log on to <https://www.editorialmanager.com/pone/> and select the 'Submissions Needing Revision' folder to locate your manuscript file.

Please include the following items when submitting your revised manuscript:

A rebuttal letter that responds to each point raised by the academic editor and reviewer(s). You should upload this letter as a separate file

labeled 'Response to Reviewers'.

A marked-up copy of your manuscript that highlights changes made to the original version. You should upload this as a separate file labeled

'Revised Manuscript with Track Changes'.

An unmarked version of your revised paper without tracked changes. You should upload this as a separate file labeled 'Manuscript'.

If you would like to make changes to your financial disclosure, please include your updated statement in your cover letter. Guidelines for resubmitting your figure files are available below the reviewer comments at the end of this letter.

If applicable, we recommend that you deposit your laboratory protocols in [protocols.io](http://protocols.io/) to enhance the reproducibility of your results. Protocols.io assigns your protocol its own identifier (DOI) so that it can be cited independently in the future. For instructions see: <https://journals.plos.org/plosone/s/submission-guidelines#loc-laboratory-protocols>. Additionally, PLOS ONE offers an option for publishing peer-reviewed Lab Protocol articles, which describe protocols hosted on [protocols.io. Read more information on sharing protocols at https://plos.org/protocols?utm_medium=editorial-email& utm_source=authorletters&utm_campaign=protocols.](https://plos.org/protocols?utm_medium=editorial-email&utm_source=authorletters&utm_campaign=protocols)

We look forward to receiving your revised manuscript.

Kind regards,

Sarah Michiels Academic Editor PLOS ONE

Response:

Dear Academic Editor, PLOS ONE, dear Dr. Sarah Michiels*,*

We would like to thank you for giving us the opportunity to submit a revised copy of our manuscript entitled ‘Physical activity, fear avoidance beliefs and level of disability in a multi-ethnic female population with chronic low back pain in Suriname: a population-based study’. We appreciate the time and effort that you and the reviewers have dedicated to providing valuable feedback on our manuscript. We are grateful to the reviewers for their insightful comments for correction or modification we believe have resulted in an improved revised manuscript. We have been able to incorporate most of the changes after reflecting on the suggestions provided by the reviewers. We have highlighted these changes within the manuscript via track changes. Please find below our responses to reviewer’s remarks, as well as the location of the changes in the revised manuscript. We look forward to hearing from you regarding our submission. We would be glad to respond to any further questions and comments that you or the reviewers may have.

Sincerely yours,

Nancy Ho-A-Tham, corresponding author

**Reviewer #1**

Comments to the Author:

Thank you for considering me to review this manuscript entitled “Physical activity, fear avoidance beliefs and level of disability in a multi-ethnic female population with chronic low back pain in Suriname: A population based study”. This study lacks novelty. The rationale is poorly justified in the introduction. Methodological details are missing. Language could be improved by editing from a native English language editor.

Response:

Thank you for your comments and useful suggestions. We have revised the abstract introduction, to make the rationale of the study clearer to the reader. We have also corrected the sentence of the objectives of the study. Please find below our responses to your remarks, as well as the location of the changes in the manuscript.

| **Reviewer’s Comments** | **Our Response** | **Location of edits with track changes in manuscript** |  |
| --- | --- | --- | --- |
| **Abstract** | | | |
| Comment 1:  Objective seems to be merged into the introduction. Kindly correct it. | Thank you for your useful suggestion! We have separated the objectives of the study from the background. The objectives have been adjusted (see also comment 4).  Adjusted and revised in the text:  **Background:** Chronic low back pain (CLBP) is an important cause for reduced daily physical activity (PA) and loss of quality of life, especially in women. In Suriname, a middle-income country in South America, the relationship between PA and CLBP is still unknown.  **Aims:** To assess the level of PA in women with CLBP of different ethnicity, and to identify whether fear avoidance beliefs (FAB), disability, co-occurring musculoskeletal pain and various sociodemographic and lifestyle factors were associated with self-reported PA. | Page 2, lines 29-36 |  |
| Comment 2:  Methods: There is no discussion of inclusion/exclusion criteria here which is imperative to include. | We have added the inclusion criteria of the study to the methods, and we have rewritten this section to make it more readable.  Inserted and revised in the text:  A cross-sectional community-based house-to-house survey was conducted between April 2016 and July 2017. The survey followed the Community Oriented Program for Control of Rheumatic Diseases methodology. Selection criteria were being female of Asian-Surinamese, African-Surinamese or of Mixed ethnicity and aged 18 or older, living in an urban area, and reporting CLBP. Data was collected on PA, FAB, disability, co-occurring musculoskeletal pain sites, CLBP intensity and sociodemographic and lifestyle factors. | Page 2, lines 37-44 |  |
| Comment 3:  Results: Confidence intervals should also be reported along with odds ratio. Interpretation of OR is incomplete without CI | We have added all the confidence intervals to the text of the abstract and to the text of the results section in the manuscript. The result section of the abstract has also been revised. Thank you for your useful suggestion.  Inserted and revised in the text:  Urban adult women with current CLBP (N=210) were selected. Nearly 57% of the population met the WHO recommendation on PA, with work-related PA as the largest contributor to total self-reported PA. Most women showed low FAB scores (FABQ-Work ≤34 (96.2%) and FABQ-PA ≤14 (57.6%)) and low disability levels (Oswestry Disability Index ≤20 (62.4%)). An inverse association between total PA and FABQ-Work (OR=0.132, CI: 0.023; 0.750) was found. In contrast, total PA had a significant, positive association with disability (OR=2.154, CI: 1.044; 4.447) and workload (OR=2.224, CI: 1.561; 3.167). All other variables showed no association with total PA.  In the main text of the manuscript the confidence intervals are also inserted in the results section:  ….FABQ-Work (OR=0.132, CI: 0.023; 0.750), which means that a higher perception of FAB is associated with lower total PA time, when compared with people with less FAB. In contrast, a significant and strong positive association between recommended WHO PA level and both the ODI score (OR=2.154, CI: 1.044; 4.447) and perceived physical workload (OR=2.223, CI: 1.561; 3.167) was found. No significant association between PA and co-MSP sites was found (Table 3).  In the domain of PA-Work, fear avoidance beliefs at work presented a significant and inverse association (OR=0.170, CI: 0.032; 0.902), which is in concordance with the results for the total level of PA. The level of pain (OR=0.402, CI: 0.164; 0.986) had an inverse association with PA-Work, while a significant and strong direct association was found with the level of disability (OR=2.404, CI: 1.198; 4.823) and perceived physical workload (OR=1.891, CI: 1.352; 2644).  Regarding PA-Leisure time domain, the level of education had a significant and high association (OR=2.099, CI: 1.030; 4.279), while age ≥60 years and NRS-11 pain scale presented with significant but low OR values (respectively OR=0.374, CI: 0.151; 0.929 and OR=0.289, CI: 0.124; 0.677), showing that age and pain have an inverse association with leisure time PA. Higher pain scores were therefore in both domains of the GPAQ a significant determinant for less PA. | Page 2, lines 45-53  Page 12, lines 294, 297, 298, 301, 302, 304, 305, 307-309 |  |
| Comment 4:  Authors are discussing associations here which was not mentioned in the objectives of the study. Results should be discussed in line with the objectives of the study. | Thank you for your useful suggestion. We have revised the aims of the study, as our main goal was indeed to explore the associations between factors such as FAB, level of disability and co-occurring musculoskeletal pain sites with level of PA. See previous comment for the revisions in the result section.  Revised in the text:  To assess the level of PA in women with CLBP of different ethnicity, and to identify whether fear avoidance beliefs (FAB), disability, co-occurring musculoskeletal pain sites and various sociodemographic and lifestyle factors were associated with self-reported PA. | Page 2, lines 33-36 |  |
| **Introduction** | | | |
| Comment 5:  This section could be improved by adding more literature pertaining to the concerned topic. Further, link between PA and other variables should be thoroughly discussed. | Thank you for the useful suggestions. The introduction has been revised (more literature added). Some paragraphs/parts have been moved and/or moved and revised.  Moved text:  PA is described by the World Health Organization (WHO) as “any bodily movement produced by skeletal muscles that requires energy expenditure” [4].  Inserted in the text:  Insufficient PA and sedentary lifestyle have been reported as risk factors for LBP, especially in older adults [3, 5, 6].  As fear avoidance beliefs (FAB) may lead to fear avoidance behavior, individuals with LBP can become fearful and avoidant of PA due to their pain experience or fear of reinjury [13].  In patients with CLBP, high FAB about work have been reported as a predictor for long-term sick leave, disability, and pain [19].  A previous study in Suriname showed that there is a high prevalence of low back pain in the urban population, especially in adult females [31]. However, little is known regarding the level of PA and its association with various factors including FAB, level of disability and sociodemographic factors in these women.  Moved and revised:  The health benefits of PA or PA-based interventions for chronic musculoskeletal pain are well documented. PA-based interventions appear to be effective in reducing pain and disability in patients with chronic musculoskeletal pain conditions [7, 8]. An inverse relationship between PA and LBP prevalence has been reported [9]. Even when CLBP is already present, moderate to vigorous leisure time PA leads to less pain and disability [10, 11]. For LBP, regular PA has a significant protective effect [10, 12]. The protective effect of leisure time PA on CLBP is seen in both men and women as well as in adults or older people. However, it has also been reported that individuals with LBP may limit their leisure time PA due to fear of pain [13]; this may progress to a vicious circle of neurological pain adaptations, and consequently lead to disability [14]. Several studies have shown various PA levels within LBP populations. In persons with acute or subacute LBP the level of PA seems to be independent of their pain-related disability [15]. While persons with CLBP and high levels of disability are likely to have low levels of PA [15, 16]. | Page 4, lines 67-68  Page 4, lines 68-70  Page 4, lines 89-91  Page 4, lines 94-95  Pages 5-6, lines 127-131  Page 4, lines 71-83 |  |
| **Methods** | | | |
| Comment 6:  Include information on psychometric properties of the outcomes variables in the methods section. | Please, see the revisions in the document. For the ODI, FABQ and pain scale the psychometric properties have been included.  Inserted and revised in the text:  Information on participants’ characteristics such as age, level of education, relationship status, perceived physical workload, LBP intensity and the presence of comorbidities were obtained using the COPCORD questionnaires [32]. To calculate the body mass index (BMI) and to assess waist circumference, weight, height, and waist circumference were measured during the visit.  Inserted in the text:  According to Bull et al [34], reliability coefficients were of moderate to substantial strength (Kappa 0.67 to 0.73; Spearman’s rho 0.67 to 0.81). Results on concurrent validity between the International Physical Activity Questionnaire, a previously validated and accepted measure of PA, and GPAQ also showed a moderate to strong positive relationship (range 0.45 to 0.65). The GPAQ demonstrated fair-to-moderate correlations (between the GPAQ and accelerometer) for moderate to vigorous PA when used in face-to-face interviews (rs = 0.46) [35].  The fear avoidance beliefs questionnaire (FABQ) was specifically developed for application in CLBP patients [39].  It has been shown to have excellent test-retest reliability (ICC=0.90 for FABQ-PA and ICC=0.96 for FABQ-Work) [42].  The ODI questionnaire has a good construct validity [44], acceptable internal consistency (Cronbach α ranges from .71 to .87) and high test-retest reliability (values range from r = 0.83 to 0.99) [45, 46].  The NRS-11 is the most widely used scale for the assessment of self-reported pain [47, 48] and it has been shown to be reliable and valid measure of pain in LBP [47-49]. | Page 7, lines 185-189  Pages 7-8, lines 191-197  Page 8, lines 210-211  Page 8, lines 216-217  Page 8, lines 222-225  Page 9, lines 229-230 |  |
| Comment 7:  Categorization of ethnic groups should be clearly mentioned. | Thank you for this comment. We have adapted this section.  Inserted, moved, and revised in the text:  In Suriname the five largest ethnic groups are represented by Hindustani (South Asian descendants mainly from India), Javanese (descendants of immigrants from Java, Indonesia), Creoles (African descendants that remained in the city during slavery), Maroons (African descendants that escaped into the hinterland during slavery), and individuals with mixed ethnicity. For this study eligible participants were categorized in Asian-Surinamese (Hindustani and Javanese), African-Surinamese (Creoles and Maroons), and Surinamese of mixed ethnicity. | Page 7, lines 170-176 |  |
| Comment 8:  Include a paragraph on study protocol where a brief summary of the study can be described. | Thank you for the useful suggestion.  Inserted and revised in the text:  In this study, the first two phases of stage I from the COPCORD methodology were conducted. In phase 1, information about sociodemographic variables (including occupation and education), presence of musculoskeletal complaints, and medical and trauma history was collected. Participants continued with phase 2 (at the same visit) if they reported any current or past musculoskeletal complaints. In phase 2 additional data regarding CLBP was collected. The original COPCORD questionnaire was translated and adapted for the local population and complemented with two standard questionnaires regarding low back pain beliefs. Both phase 1 and 2 questionnaires were administered by interviewers trained by a rheumatologist (RW). The target number of household addresses (N=1125) in our study was based on an average number of four respondents per household and on population proportions from the Census data of 2012. A random sample of household addresses within different areas of the two districts were selected via a stratified, multi-stage, cluster, sampling design as described by Krishnadath et al [33]. A door-to-door survey was conducted afterwards. Maximum three attempts were made to interview each eligible household member.  The data used in the study is from a parent (COPCORD Suriname) survey, with a total population of 2221 [32]. For the present study we included all women from the COPCORD urban study population who reported experiencing CLBP (i.e., pain, muscle tension or stiffness between the costal margin and the lower gluteal folds for 12 weeks or longer), who were ≥18 years and of Asian-Surinamese, African-Surinamese or of Mixed ethnicity. Written informed consent was obtained from all participants and confidentiality of participants was strictly maintained. | Pages 6-7, lines 146-166 |  |
| Comment 9:  Statistical analysis:  Some points regarding the data analysis are unclear. Intricate details of regression analysis are required. What were the predictors and independent variables in the analysis? What all covariates were considered in the regression analysis? | Thank you for the useful suggestion.  We have added more information to the methods, especially about the regression models.  Inserted and revised in the text:  To identify significant determinants associated with PA and its specific domains (work-related and leisure time), logistic regression models were constructed. The first model included total self-reported PA as dependent variable. The second model included PA Work domain as dependent variable and the third model PA Leisure time domain. All three models included all other variables as independent variables. Covariates such as age, relationship status and education were also used in the three models. Validated cut-off points were used to dichotomize variables.  Deleted:  In the current study only PA during work and leisure time PA were used in the regression.  Binomial logistic regression models were constructed… | Page 9, lines 241-249  Page 9, lines 239-240  Page 9, line 241 |  |
| **Discussion** | | | |
| Comment 10:  Line 252-254: Age and educational level were not variables of interest. Results should be discussed in line with the objectives of the study.  Discussion seems insufficient. Findings of all variables are not discussed and relevant studies are very scant. A detailed discussion on findings of each variable is required. | The aims of the study have been adjusted (see previous comments). The discussion section has been revised. Relevant studies have been added.  In the results section the following text have been adjusted to be in line with the objectives of the study.  Moved to:  Most of the women in the present study showed low FAB due to CLBP, reporting scores below the cutoff point for both the FABQ-Work ≤34 (96.2%) and FABQ-PA ≤14 (57.6%) subscales (Table 1).  Deleted:  …and fear avoidance beliefs  Summary of main findings  Physical activity, physical activity domains and chronic low back pain  Association between physical activity and fear avoidance beliefs  Inserted and revised:  …associations between PA and FAB, level of disability, co-MSP, various sociodemographic and lifestyle factors.  …. total self-reported PA and heavy workload, FABQ-Work and moderate to severe disability. Other variables such as co-MSP sites, the level of pain, ethnicity, comorbidities, sociodemographic (age, education, marital status) and lifestyle factors (BMI and waist circumference, smoking, workload, and work activities) showed no association with total PA in this study population.  According to the South American physical activity and sedentary behavior network which also used the GPAQ, Chile and Brazil, reported 51.1% and 52.4% respectively, for PA prevalence in women [51]. Similar prevalence rate for PA was reported for urban women in Ethiopia (50.6%) [52].  In our study, a positive relationship between educational level and PA-Leisure time was reported. Other studies also showed that higher educated individuals were more physically active during leisure-time [6, 60-62]. Persons with a higher educational level might have better resources to incorporate PA in daily lives [62]. In lower educated persons, a possible explanation might be low perceived control of life, an important predictor for decreasing PA-Leisure time, in lower educated persons [61]. Age was found to be significantly and inversely associated with PA-Leisure time. This association is also found in persons without CLBP [11]. Pain was also a significant determinant for less PA in both PA-Work as in PA-Leisure time. These outcomes are in concordance with the literature [11, 55, 63].  Inserted in the text:  In low- and middle-income countries, facilitators for PA may be lacking and social, structural, and individual barriers could be reasons why individuals do not participate in leisure-time PA [56, 57].  In the present study, average to heavy workload was associated with total PA and PA-work. High workload has been reported as risk for future LBP [54, 58, 59].  …. aspects of work such as workload and the type of work activities should be considered when evaluating PA in the context of CLBP management.  Our study was not in agreement with previous studies investigating the association with PA and disability in CLBP [15, 16]. De Sousa et al [15] reported an inverse relationship between PA and disability in CLBP patients in Brazil. A systematic review by Lin et al [16] also reported these same results. In the present study, moderate to severe disability was positively associated with total self-reported PA and PA-work. This means that participants in our study were able to maintain a certain level of PA during work despite reporting moderate to severe levels of disability. Probably, different patterns of PA such as type and quality of PA may differ [64], social desirability leading to overreporting of PA [65] and heterogeneity in the definitions of PA and CLBP may have led to these conflicting results.  As confusing evidence still exist regarding the relationship between PA and CLBP for CLBP management, sedentary behavior might be equally important to pay attention to. Sedentary behavior is defined as ‘any waking behaviors characterized by an energy expenditure ≤1.5 METs, while in a sitting, reclining, or lying posture’ [71]. According to Thivel et al [72] a person can be classified as both (in)active and sedentary. In a recent systematic review by Alzahrani et al [73] sedentary behavior seems to play an important role in the association between CLBP and poor disability. No associations were found between sedentary behavior and LBP risk or pain intensity. This was conflicting evidence with previous findings reporting an association between PA and LBP risk [6, 10, 11]. A combination of sedentary behavior with low levels of PA may increase LBP risk [3]. As PA during work activities contributed largely to the total PA score in our study, it is necessary to include determinants of sedentary behavior during work to develop treatment strategies, especially in occupational health. Sedentary behavior was not within the scope of this study. Future research is needed to elaborate on this topic.  Literature confirms these findings between PA and FAB [27, 70, 74]. In a recent study by Naugle et al [75] this inverse association between PA and fear of movement was also seen in chronic pain-free older adults. Various studies, however, reported no association between PA and FAB [28, 29, 76]. Reasons for these discrepancies can lie in the use of different assessment instruments for PA and FAB. Although FABQ showed good psychometric properties, it does not define the specific activities that are feared or avoided. Leonhardt et al [29] also stated that possibly the quality and not the quantity of certain activities are associated with FAB.  It is important that healthcare professionals should be aware of fear avoidance beliefs and behaviors, as it can potentially reduce PA and increase the risk of disability. These maladaptive beliefs together with pain intensity and pain catastrophizing have been reported as important determinants of disability [19, 79]. Recognition of these beliefs are also paramount in the acute or sub-acute phase of LBP, as these unhelpful beliefs can increase the likelihood of delayed recovery and chronicity [80]. Additionally, a reduction in FAB at the early stages of LBP may lead to an increase of activities in daily and social life [79]. | Page 10, lines 261-264  Page 11, line 274  Page 14, line 317  Page 14, line 329  Page 16, line 402  Page 14, lines 320-321  Page 14, lines 324-328  Page 14, lines 334-337  Page 15, lines 359-367  Page 14, lines 344-346  Page 15, lines 353-354  Page 15, lines 356-358  Page 15, lines 368-376  Page 16, lines 388-401  Page 16, lines 406-413  Page 17, lines 421-427 |  |

| **Reviewer #2** |  |  |
| --- | --- | --- |
| Comments to the Author:  Reviewer #2: thank you for your effort, and your manuscript needs some revision  The purpose of this study is to examine the physical activity, fear avoidance beliefs and level of disability in a multi-ethnic female population with chronic low back pain in Suriname. The statistical power and research design are sound. | | |
| Response: We would like to thank you for your useful comments and suggestions. We appreciate your support. Please find below our changes. | | |
| **Abstract** | | |
| Comment 1:  In abstract introduction: Chronic low back pain (CBLP) change to CLBP | Thank you for your comment. See the correction in the text.  (CBLP) has been changed to (CLBP) | Page 2, line 29 |
| **Introduction** | | |
| Comment 2:  Understanding the rationale for the research question can be improved through a revised introduction. | Thank you for the useful suggestions. The introduction has been revised. The sequence of some sections has been changed.  Moved text:  PA is described by the World Health Organization (WHO) as “any bodily movement produced by skeletal muscles that requires energy expenditure” [4].  Inserted in the text:  Insufficient PA and sedentary lifestyle have been reported as risk factors for LBP, especially in older adults [3, 5, 6].  As fear avoidance beliefs (FAB) may lead to fear avoidance behavior, individuals with LBP can become fearful and avoidant of PA due to their pain experience or fear of reinjury [13].  In patients with CLBP, high FAB about work have been reported as a predictor for long-term sick leave, disability, and pain [19].  A previous study in Suriname showed that there is a high prevalence of low back pain in the urban population, especially in adult females [31]. However, little is known regarding the level of PA and its association with various factors including FAB, level of disability and sociodemographic factors in these women.  Moved and revised:  The health benefits of PA or PA-based interventions for chronic musculoskeletal pain are well documented. PA-based interventions appear to be effective in reducing pain and disability in patients with chronic musculoskeletal pain conditions [7, 8]. An inverse relationship between PA and LBP prevalence has been reported [9]. Even when CLBP is already present, moderate to vigorous leisure time PA leads to less pain and disability [10, 11]. For LBP, regular PA has a significant protective effect [10, 12]. The protective effect of leisure time PA on CLBP is seen in both men and women as well as in adults or older people. However, it has also been reported that individuals with LBP may limit their leisure time PA due to fear of pain [13]; this may progress to a vicious circle of neurological pain adaptations, and consequently lead to disability [14]. Several studies have shown various PA levels within LBP populations. In persons with acute or subacute LBP the level of PA seems to be independent of their pain-related disability [15]. While persons with CLBP and high levels of disability are likely to have low levels of PA [15, 16]. | Page 4, lines 67-68  Page 4, lines 68-70  Page 4, lines 89-91  Page 4, lines 94-95  Pages 5-6, lines 126-130  Page 4, lines 71-83 |
| **Discussion** |  |  |
| Comment 3:  This section needs more explanation particularly, comparisons with previous similar studies. | The aims of the study have been adjusted (see previous comments). The discussion section has been revised. Relevant studies have been added.  Inserted and revised:  …associations between PA and FAB, level of disability, co-MSP, various sociodemographic and lifestyle factors.  …. total self-reported PA and heavy workload, FABQ-Work and moderate to severe disability. Other variables such as co-MSP sites, the level of pain, ethnicity, comorbidities, sociodemographic (age, education, marital status) and lifestyle factors (BMI and waist circumference, smoking, workload, and work activities) showed no association with total PA in this study population.  According to the South American physical activity and sedentary behavior network which also used the GPAQ, Chile and Brazil, reported 51.1% and 52.4% respectively, for PA prevalence in women [51]. Similar prevalence rate for PA was reported for urban women in Ethiopia (50.6%) [52].  In our study, a positive relationship between educational level and PA-Leisure time was reported. Other studies also showed that higher educated individuals were more physically active during leisure-time [6, 60-62]. Persons with a higher educational level might have better resources to incorporate PA in daily lives [62]. In lower educated persons, a possible explanation might be low perceived control of life, an important predictor for decreasing PA-Leisure time, in lower educated persons [61]. Age was found to be significantly and inversely associated with PA-Leisure time. This association is also found in persons without CLBP [11]. Pain was also a significant determinant for less PA in both PA-Work as in PA-Leisure time. These outcomes are in concordance with the literature [11, 55, 63].  Inserted in the text:  In low- and middle-income countries facilitators for PA maybe lacking and social, structural, and individual barriers could be reasons why individuals do not participate in leisure-time PA [56, 57].  In the present study, average to heavy workload was associated with total PA and PA-work. High workload has been reported as risk for future LBP [54, 58, 59].  …. aspects of work such as workload and the type of work activities should be considered when evaluating PA in the context of CLBP management.  Our study was not in agreement with previous studies investigating the association with PA and disability in CLBP [15, 16]. De Sousa et al [15] reported an inverse relationship between PA and disability in CLBP patients in Brazil. A systematic review by Lin et al [16] also reported these same results. In the present study, moderate to severe disability was positively associated with total self-reported PA and PA-work. This means that participants in our study were able to maintain a certain level of PA during work despite reporting moderate to severe levels of disability. Probably, different patterns of PA such as type and quality of PA may differ [64], social desirability leading to overreporting of PA [65] and heterogeneity in the definitions of PA and CLBP may have led to these conflicting results.  As confusing evidence still exist regarding the relationship between PA and CLBP for CLBP management, sedentary behavior might be equally important to pay attention to. Sedentary behavior is defined as ‘any waking behaviors characterized by an energy expenditure ≤1.5 METs, while in a sitting, reclining, or lying posture’ [71]. According to Thivel et al [72] a person can be classified as both (in)active and sedentary. In a recent systematic review by Alzahrani et al [73] sedentary behavior seems to play an important role in the association between CLBP and poor disability. No associations were found between sedentary behavior and LBP risk or pain intensity. This was conflicting evidence with previous findings reporting an association between PA and LBP risk [6, 10, 11]. A combination of sedentary behavior with low levels of PA may increase LBP risk [3]. As PA during work activities contributed largely to the total PA score in our study, it is necessary to include determinants of sedentary behavior during work to develop treatment strategies, especially in occupational health. Sedentary behavior was not within the scope of this study. Future research is needed to elaborate on this topic.  Literature confirms these findings between PA and FAB [27, 70, 74]. In a recent study by Naugle et al [75] this inverse association between PA and fear of movement was also seen in chronic pain-free older adults. Various studies, however, reported no association between PA and FAB [28, 29, 76]. Reasons for these discrepancies can lie in the use of different assessment instruments for PA and FAB. Although FABQ showed good psychometric properties, it does not define the specific activities that are feared or avoided. Leonhardt et al [29] also stated that possibly the quality and not the quantity of certain activities are associated with FAB.  It is important that healthcare professionals should be aware of fear avoidance beliefs and behaviors, as it can potentially reduce PA and increase the risk of disability. These maladaptive beliefs together with pain intensity and pain catastrophizing have been reported as important determinants of disability [19, 79]. Recognition of these beliefs are also paramount in the acute or sub-acute phase of LBP, as these unhelpful beliefs can increase the likelihood of delayed recovery and chronicity [80]. Additionally, a reduction in FAB at the early stages of LBP may lead to an increase of activities in daily and social life [79]. | Page 14, lines 320-321  Page 14, lines 324-328  Page 14, lines 334-337  Page 15, lines 359-367  Page 14, lines 344-346  Page 15, lines 353-354  Page 15, lines 356-358  Page 15, lines 368-376  Page 16, lines 388-401  Page 16, lines 406-413  Page 17, lines 421-427 |
|  |  |  |
